# Lipid Metabolism Reprogramming in Tumor-Associated Macrophages Modulates Their Function in Primary Liver Cancers

**DOI:** 10.3390/cancers17111858

**Published:** 2025-05-31

**Authors:** Barbara Oliviero, Anna Caretti, Mario U. Mondelli, Stefania Mantovani

**Affiliations:** 1 Department of Translational and Clinical Research, Division of Molecular Medicine, Laboratory of Clinical Immunology, Fondazione IRCCS Policlinico San Matteo, 27100 Pavia, Italy; b.oliviero@smatteo.pv.it (B.O.); s.mantovani@smatteo.pv.it (S.M.); 2 Department of Health Sciences, University of Milan, 20142 Milan, Italy; anna.caretti@unimi.it

**Keywords:** hepatocellular carcinoma, cholangiocarcinoma, macrophages, lipids, metabolism, immunosuppression, tumor microenvironment

## Abstract

Lipids are a complex class of biomolecules with pivotal roles in the onset, progression, and maintenance of cancers. Lipids, derived from the tumor microenvironment (TME) or synthesized by cancer cells themselves, govern a large variety of pro-tumorigenic functions. In recent years, lipid metabolism and the reprogramming of liver cancer cells have received increasing attention, revealing that altered regulation of diverse lipid species, including triacylglycerols, phospholipids, sphingolipids, ceramides, fatty acids, and cholesterol, actively contributes to the initiation and progression of primary liver cancer. Lipid metabolic reprogramming also modifies the TME by influencing the recruitment, activation, and function of immune cells. Tumor-associated macrophages (TAM) are essential components of TME that sustain cancer growth, promoting invasion and mediating immune evasion. Macrophage polarization toward a tumor-supportive phenotype is associated with metabolic reprogramming. Indeed, lipid accumulation and enhanced fatty acid oxidation in TAM contribute to polarization to a M2 phenotype. In this review, we examine lipid metabolism in hepatocellular carcinoma and cholangiocarcinoma, focusing on TAM lipid metabolic reprogramming.

## Simple Summary

Lipids play a key role in the onset, progression, and maintenance of cancers. Lipids from the tumor’s surroundings or synthesized by cancer cells govern many processes that help tumors grow. In addition to supporting tumor development, lipids modify the tumor microenvironment by influencing the recruitment, activation, and function of many immune cells, especially the tumor-associated macrophages. Indeed, macrophages infiltrating the tumor are essential to sustain cancer growth, promoting invasion and mediating immune evasion. This article seeks to review the current research concerning lipid metabolism in the two most frequent primary liver tumors, namely hepatocellular carcinoma and cholangiocarcinoma, focusing on pathways that modify the phenotype and function of tumor-associated macrophages.

## 1. Introduction

Globally, primary liver cancer ranks as the sixth most frequently diagnosed cancer and the third most common cause of cancer mortality [1]. The two main types of primary liver cancer are hepatocellular carcinoma (HCC) (75–85%) and intrahepatic cholangiocarcinoma (iCCA) (10–15%) [2].

The development of HCC occurs in the setting of chronic liver diseases, including viral hepatitis, alcoholic liver disease, and metabolic dysfunction-associated steatotic liver disease (MASLD), via a stepwise progression marked by sustained inflammation, tissue necrosis, and cirrhosis [3]. Treatment options for HCC include locoregional therapies, surgical approaches (resection or liver transplantation), as well as systemic treatments such as chemotherapy and immunotherapeutic agents. Despite significant progress in therapies over the years, HCC still frequently develops drug resistance, recurs, metastasizes, and carries an unfavorable prognosis [4].

Unlike HCC, which nearly always develops in cirrhotic livers, iCCA typically occurs in livers without pre-existing cirrhosis. Because early-stage CCA typically causes no symptoms and lacks reliable biomarkers, approximately 60% of patients are diagnosed at advanced stages when curative options like surgical resection or transplantation are no longer feasible, leading to poor outcomes [5]. Furthermore, the remarkable heterogeneity in both pathological features and gene expression patterns contributes to the notably poor efficacy of radiotherapy and chemotherapy in CCA treatment [6,7]. Therefore, given the modest benefit of the current therapeutic approaches, it is mandatory to identify new pathways and molecules that could represent novel diagnostic tools or therapeutic targets in primary liver cancer treatment.

The metabolic reprogramming characteristic of tumor cells drives a cascade of molecular changes enabling escape from immune surveillance [8]. Multiple studies [9,10] have demonstrated significant lipid metabolic alterations in both HCC and iCCA, underscoring the pathogenic role of lipid dysregulation in liver carcinogenesis and progression [11,12]. Currently, lipids are fundamentally involved in cancer pathobiology, driving tumor initiation, progression, and maintenance. Beyond regulating tumor cell biology, accumulating evidence demonstrates that lipids actively modulate the function and status of immune cells within the tumor microenvironment (TME) [13]. Through active release of signaling molecules and metabolites, neoplastic cells orchestrate extensive modifications of the TME that alter non-transformed cell behavior [14]. Simultaneously, lipid metabolic remodeling in non-tumor cells drives the environment toward an immunosuppressive phenotype that facilitates malignant progression [15]. Tumor-derived lipids within the TME actively regulate immune cell recruitment, activation states, and effector functions. In particular, the accrual of lipids within tumor-associated macrophages (TAM) contributes to their reprogramming towards an immunosuppressive phenotype that facilitates malignant growth [16,17].

## 2. Lipid Metabolism in Primary Liver Cancers and Therapeutic Perspectives

Lipids are a complex class of biomolecules with pivotal roles in membrane construction, signal transduction, and cellular energy fueling.

MASLD represents a spectrum of metabolic liver disorders linked to obesity, progressing from simple hepatic steatosis to the more severe metabolic dysfunction-associated steatohepatitis (MASH). An estimated 20% of MASLD patients develop MASH, with a proportion of these cases subsequently progressing to MASH-related HCC [18]. In MASLD/MASH, hepatic lipid accumulation results from both increased influx and de novo synthesis combined with defective lipid oxidation and/or export mechanisms. Carli et al. performed an extensive analysis of lipid metabolism’s role in MASLD/MASH progression, particularly examining weight loss-induced changes in hepatic lipid metabolism, lipidomic signatures, and MASH resolution [19].

Dysregulation of hepatic lipid metabolism has been associated with tumor development and progression in patients with HCC and CCA [20,21]. Lipid synthesis activation triggers inflammation [22], oxidative stress [23,24] and lipotoxicity [25] promoting hepatocarcinogenesis.

Numerous studies have described relevant alterations in the fatty acid (FA) profile of liver cancers, particularly a reduction in polyunsaturated fatty acids (PUFAs) and an increase in monounsaturated and saturated fatty acids (MUFAs) [26,27,28], which support cancer cell proliferation, migration, and immune suppression. Considering first CCA, this tumor seems to be dependent on exogenous FA uptake rather than on de novo FA biosynthesis. In the liver, FATP2, 3 and 5 (FA transport proteins), FABP 1, 4 and 5 (FA binding proteins) and the translocase CD36 protein actively transport FA through cell membranes [29]. Of note, FABP5 over-expression correlates with a worse prognosis in extrahepatic compared with iCCA [30]. Indeed, the silencing of specific FA transporters in CCA cell lines leads to a decrease in cell growth [31]. Accordingly, in AKT/Ras mice developing HCC and iCCA, only the development of HCC was affected by FA synthase (FASN) knockdown [32,33]. However, we performed an in-depth lipidomic analysis of tumor and non-tumor surrounding tissues in iCCA patients, as well as of patients’ and healthy controls’ sera [10], demonstrating that newly synthesized FA accumulated in iCCA and were addressed to membrane-forming phospholipids and sphingomyelins. Of note, FA were poorly directed to mitochondria β-oxidation or lipid droplets (LD) deposition as energy-reservoir neutral species, while supporting macrophage M2 phenotype. In HCC, different studies have demonstrated that enhanced de novo FA synthesis and suppression of FA oxidation (FAO) contribute to tumorigenesis [34,35,36,37]. Despite a wide consensus on FA synthesis upregulation in HCC, findings regarding FAO are far from consistent. As recently reviewed [38], HCC patients with enhanced β-catenin signaling activate FA catabolism rather than relying on the Warburg effect to sustain energy demand. In contrast, HCC patients with hepatic steatosis avoid FA catabolism to support tumor progression. Further studies highlighted contradictory findings about FAO. In HCC cells, FAO inhibition likely protects tumor cells from oxidative stress and lipotoxic death [39,40], though some authors show that FAO promotion supports tumor proliferation and drug resistance and prevents energy starvation-induced cell death [41,42].

Glycerophospholipid (GPL) reprogramming is a common mechanism driving cancer progression in HCC and CCA. Untargeted lipidomic analysis revealed significant upregulation of phosphatidylcholine and lysophosphatidylcholine within the GPL pathway and overexpression of PLA2, a key enzyme in GPL metabolism [43]. In HCC, the altered homeostasis of GPL is likely involved in resistance to ferroptosis, the stimulation of cell proliferation, and migration, as well as immunosuppression [44]. Among GPL, MUFA-phosphatidylcholines accumulation in HCC is associated with hepatocyte proliferative stimulus that triggers HCC onset [22].

Sphingolipids are an important lipid class that is upregulated in both HCC [45,46] and CCA [47]. Indeed, the accumulation of sphingosine 1-phosphate (S1P) promotes tumor development and epithelial-to-mesenchymal transition in HCC [48,49], as well as metastasis in CCA [48]. Low ceramide and high sphingomyelin levels were detected in HCC compared with non-tumor surrounding tissues [50]. Recently, we examined lipid dysmetabolism in iCCA. Via liquid chromatography-tandem mass spectrometry (LC–MS/MS) analysis, we characterized sphingolipid content in primary iCCA cell-derived extracellular vesicles (EV) and showed an enrichment of ceramide and dihydroceramides in poorly differentiated iCCA-EV [51].

In the liver, cholesterol is metabolized to bile acids. It has long been known that a high cholesterol content is typical of HCC compared with healthy liver tissue [52], though the underlying mechanisms vary between different liver tumors and are not fully clarified yet. In HCC, Sharif et al. [53] reported higher hepatic bile acid (BA) levels, which contribute to HCC pathogenesis by increasing oxidative stress, inflammation [54], and cell invasion [55]. CCA exhibits cholesterol-derived BA accrual that may prompt abnormal cell proliferation and development [56]. Moreover, BAs and conjugated BAs stimulate CCA invasiveness and inhibit apoptosis through activation of sphingosine 1-phosphate receptor 2 [57].

Cancer stem cells (CSC) are involved in the maintenance of malignant characteristics of many solid tumors, including liver cancer. Within HCC, massive upregulation of acetyl-coA that is transformed into enhanced lipid content impacts liver CSC properties, such as self-renewal, differentiation, invasion, metastasis, and drug sensitivity [58]. Raggi C. and colleagues [11] provide evidence for the activation of the FA metabolism, which confers a stem-like phenotype in iCCA. They suggest that the de novo synthesis and desaturation of FA may be essential in sustaining the cell pluripotency that drives iCCA cell reprogramming.

**Figure 1:**
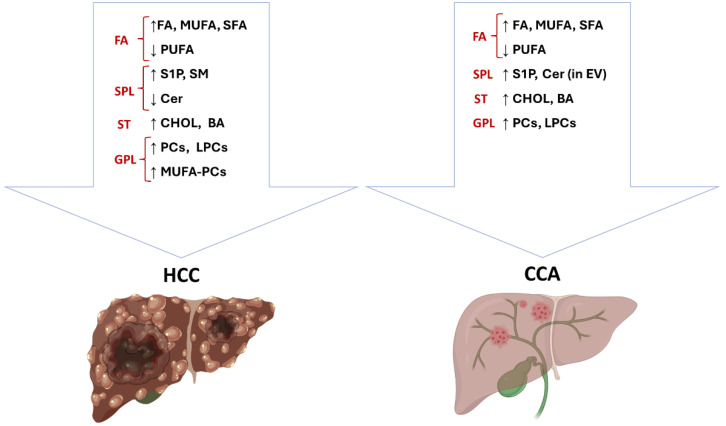
Lipid content is highly deregulated in primary liver cancer. The figure summarizes lipid alterations in HCC and iCCA as discussed in the text. BA: bile acids; Cer: ceramides; CHOL: cholesterol; FA: fatty acids; GPL: glycerophospholipids; LPCs: lyso-phosphatidylcholines; MUFA: mono-unsaturated fatty acids; MUFA-PCs: mono-unsaturated fatty acids phosphatidylcholines; PCs: phosphatidylcholines; PUFA: poly-unsaturated fatty acids; S1P: sphingosine -1 phosphate; SFA: saturated fatty acids; SM: sphingomyelins; SPL: sphingolipids; ST: sterols. This presentation was created by BioRender (version 201).

Lipid metabolism deregulation is a hallmark of primary liver cancer (Figure 1), and different compounds have been tested mostly in preclinical models targeting crucial steps in the lipid pathway in a therapeutic perspective.

Sterol regulatory element-binding proteins (SREBPs) play a crucial role in lipogenesis. Inhibiting protein expression [59,60] and activity [61] reduces HCC progression. In CCA cell lines, the nucleoside antibiotic cordycepin inhibits SREBP1-mediated FA synthesis and blocks metastasis and the epithelial-to-mesenchymal transition [62].

The main enzymes involved in lipogenesis, FASN and acetyl-CoA carboxylase (ACC), are upregulated and are often related to poor outcomes in advanced HCC stages [32,63]. Indeed, genetic ablation of FASN in mice impairs HCC progression driven by AKT [64].

Various FA synthesis inhibitors towards FASN proved efficacious in slowing HCC progression in cell and animal models. Among these, TVB-3664—alone or in combination with the tyrosine kinase inhibitors cabozantinib or sorafenib—downregulates multiple cancer-related pathways in an HCC mouse model and HCC cell lines [65]. However, as recently reported by Terry A.R. [66], HCC patients are not included in the six ongoing clinical trials for FASN inhibition in cancer. As for CCA, the role of FASN is still controversial since enhanced FA uptake also has a great impact on tumor progression. Of note, among the new FASN inhibitors, TVB-2640 is already in clinical trials for the treatment of MASLD, which represents an elevated risk of iCCA [67].

By means of multi-omic approaches, it was found that in HCC, the aberrant overexpression of TRIM45, a critical E3 3 ubiquitin ligase, amplifies peroxisome proliferator-activated receptor gamma (PPARγ)-regulated transcription of downstream FA synthesis genes [68]. The natural flavonoid Oroxyloside (OAG) is a dual PPARγ/ɑ agonist that promotes HCC cell cycle arrest via glycolipid metabolism switch mediated by ROS overproduction [69]. Increasing research correlates PPARs signaling and HCC occurrence, but there is no consensus among researchers regarding the therapeutic effects of PPARs agonists or antagonists on HCC [70]. A phase I, open-label, dose-escalation study (NCT03829436) has been conducted to test the clinical activity of TPST-1120, a first-in-class oral inhibitor of PPARα, in patients with advanced renal cell carcinoma (RCC) and CCA. TPST-1120 was well tolerated and showed preliminary evidence of efficacy in these immune-compromised cancers [71].

In HCC patients exhibiting high mTORC2 signaling, tumorigenesis is enhanced by the activation of FA and lipids synthesis [45]. Everolimus (RAD001), Deforolimus (AP23573), and Temsirolimus (CCI-779) are among the first-generation mTOR inhibitors derived from Rapamycin, referred to as Rapamycin analogs or Rapalogs. As was reviewed in-depth by Xinjun Lu et al., preclinical studies highlight the therapeutic potential of these compounds in modulating HCC growth in both colony formation assay and xenograft models. Different clinical trials for advanced HCC treatment with Rapalogs have shown preliminary evidence of clinical activity though the results obtained as single agent were almost unsatisfactory [72]. Clinical trials evaluating the clinical efficacy of everolimus, an mTORC1 inhibitor, in patients with advanced CCA reported contrasting results as a monotherapy or in combination [11].

The overexpression of the sphingosine kinases that are responsible for S1P biosynthesis correlates with reduced HCC and CCA patient survival [73,74], as well as HCC drug resistance [73]. Patients with advanced CCA partially responded to S1P inhibitor ABC294640 treatment in a Phase I clinical trial [75], while a Phase I/IIA trial in iCCA (NCT03377179) and a Phase II trial in HCC (NCT02939807) are ongoing.

Hypercholesterolemia is usually controlled by inhibiting HMG-CoA reductase activity with statins. Rosuvastatin prevents HCC development in mice [76], and fluvastatin combined with the multi-kinase inhibitor sorafenib controls cancer cell proliferation and triggers apoptosis [77]. In a randomized controlled trial, patients affected by advanced HCC showed significant prolonged survival following pravastatin administration compared to the untreated ones [78]. In large cohort studies [79,80] statin therapy reduces the risk of CCA. In preclinical models, statins induce CCA cell cycle arrest and apoptosis [81,82].

Dysregulation of lipid metabolism in primary liver cancers involves multiple pathways and molecules, including several membrane lipid transporters. This likely perturbs the lipid content of the TME, which may impact lipid fruition by other cells, ultimately influencing the behavior of immune cells.

## 3. Liver Macrophages

As the predominant immune population in the liver [83], macrophages comprise two distinct populations: (1) self-renewing, long-lived resident Kupffer cells (KC), and (2) rapidly recruited bone marrow-derived monocytes (MoMFs), which represent the “emergency response team” [84,85,86]. Advanced techniques, such as cell tracking, multi-omics phenotyping, single-cell RNA sequencing (scRNA-seq), and spatial transcriptomics, have uncovered previously unrecognized heterogeneity in liver macrophage origins and functions and defined their spatial location in the healthy liver [85,87,88]. Using neurologically deceased healthy donor (NDD) livers as reference specimens, researchers constructed a liver atlas, revealing two predominant macrophage populations that exhibit opposing relationships with inflammation. One CD68+ macrophage cluster, characterized by enriched expression of LYZ, CSTA, and CD74, represents inflammatory macrophages, and a further CD68+ macrophage cluster expresses genes, such as VSIG4 and HMOX1, related to tolerogenic function, suggesting that these macrophages have immunoregulatory phenotypes [87]. This study was recently expanded to reveal additional macrophage diversity. Ten distinct macrophage phenotypes were identified that were conserved across all donors, of which the non-inflammatory KC and inflammatory MoMFs accounted for the majority (40%) of all macrophages in the NDD liver. The remaining cells were distributed among eight less frequent phenotypes. Notably, the study uncovered a distinct macrophage subpopulation marked by the expression of PLAC8, LST1, IFITM3, AIF1, and COTL1, along with inflammatory markers (FCN1, LYZ, S100A4, S100A8). Pathway analysis of this subset demonstrated significant enrichment in interferon (IFN)-alpha response, TCR signaling, immunological synapse formation, and IL8-CXCR2 axis pathways, implying potential crosstalk with T lymphocytes. Spatial transcriptomics identified a distinct spatial location of different macrophage populations, suggesting the association between macrophage functional/phenotype with their distinct location within the microenvironment [88].

It is evident that liver macrophages exhibit remarkable heterogeneity in terms of phenotype, function, and localization, reflecting a broad spectrum of potential interactions with other immune cells, as well as multiple responses to tissue damage.

## 4. TAM Diversity in Primary Liver Cancer

The complex TME comprises diverse non-neoplastic cellular components, including both innate and adaptive immune cells that critically influence tumor progression and metastatic dissemination. TAMs, constituting approximately half of all hematopoietic cells in the TME, have been extensively characterized as key modulators of this complex ecosystem [89]. TAMs exhibit remarkable functional plasticity, capable of both pro-tumorigenic activities (supporting cancer cell survival, proliferation, angiogenesis, and metastasis), anti-tumor effector functions (mediating antibody-dependent cellular cytotoxicity, phagocytosis, and vascular damage), and activating anti-tumor adaptive immune responses [90,91,92]. Current TAM characterization has been limited by inadequate functional markers. While CD68 and CD163 are commonly used to characterize macrophages, these surface markers fail to capture the functional diversity of TAMs. Both MoMFs and tissue resident macrophages exhibit remarkable phenotypic plasticity in response to minimum TME variations, including nutrient gradients, metabolic alterations, and hypoxic conditions. This adaptability generates substantial TAM heterogeneity across cancer types and within the same tumor [93]. The classical macrophage classification system delineates two primary subsets: M1 macrophages, activated by IFN-γ and TLR ligands (e.g., LPS), and M2 macrophages, which exhibit heterogeneous subtypes defined by exposure to distinct molecular signals [92,93,94]. TAMs exhibit a dichotomous polarization state: M1-like (CD68+ IL-1β+) macrophages can act as primary mediators of innate host defense, while M2-like (CD163+ IL-10+ CCL18+) macrophages promote epithelial-mesenchymal transition, angiogenesis, and tumor immunosuppression [95,96,97]. The M1/M2 classification is now seen as oversimplified, given the spectrum of intermediate macrophage phenotypes observed in different pathologies or in response to varying stimuli [85]. The use of single-cell technologies and cytometry by time-of-flight (CyTOF) enables the identification of novel TAM subpopulations with unique functional profiles. Emerging evidence from various solid tumors suggests a revised TAM classification comprising four subsets distinguished by core transcriptional programs, with the C1Q+ and SPP1+ TAMs representing the dominant populations and the FCN1+ and CCL18+ TAMs appearing less frequently [98].

With respect to HCC, multiple single-cell studies have shed light on the phenotype and functional characteristics of TAMs. Thus, both M1- and M2-like signatures were found to coexist within the TME of patients with HBV-associated HCC, highlighting the greater complexity of TAMs in HCC beyond the classical M1/M2 paradigm [99]. Six macrophage clusters were identified in the dataset, with THBS1+ and C1QA+ subsets being particularly enriched in tumor tissues. The C1QA+ cluster exhibited gene expression signatures characteristic of both M1- and M2-like macrophages. Transcriptomic profiling revealed that THBS1+ and C1QA+ macrophages formed a continuum while maintaining distinct gene expression profiles. Interestingly, C1QA+ macrophages showed elevated expression of SLC40A1 (ferroportin), which regulates iron export and modulates TLR-mediated production of IL-6, IL-23, and IL-1beta, along with GPNMB. Based on these data, the authors surmised that iron metabolism was involved in polarizing macrophage phenotype in the TME, shaping innate immunity in HCC [99]. A THBS1⁺ myeloid cell population, expressing genes associated with monocytes and neutrophils and characterized by the co-expression of TREM1 and CD163, was identified as enriched in tumors from patients with steatotic liver disease-associated HCC. These cells, termed THBS1⁺ regulatory myeloid (Mreg) cells, demonstrated strong suppression of T cell activity ex vivo, a function that was further enhanced upon TREM1 activation. Mreg cells were enriched in fibrotic lesions of HCC and co-localized with FAP⁺ cancer-associated fibroblasts (CAF), as demonstrated by spatial transcriptomics RNA-seq. The intratumoral density of THBS1+ Mreg and the median expression of TREM1 were both correlated with high-grade HCC and poor patient prognosis [100].

In patients with HBV-associated HCC, various cellular clusters expressing immunosuppressive molecules were identified, overlapping with the enrichment pattern of CD163, a marker of M2 macrophages. In these patients, the abundance of cancer-promoting, anti-inflammatory M2-like TAMs was inversely correlated with tumor-infiltrating lymphocytes, suggesting that TAMs may inhibit T-cell infiltration [101]. In a separate study analyzing patients with primary and early relapsed HBV-associated HCC, researchers found that conventional M1/M2 classification markers (FCGR3A for M1, CD163 for M2) failed to clearly distinguish macrophage polarization states [102]. However, by assessing M1 and M2 polarization alongside pro- and anti-inflammatory scores based on relevant gene sets, a predominantly M2-like phenotype was identified. Through in vitro experiments and a mouse model, the role of APOC1 in TAMs in human HCC of various etiologies was established [103]. APOC1 expression was significantly elevated in intratumoral macrophages relative to their counterparts in adjacent liver tissue. Inhibition of APOC1 induces a phenotypic shift from pro-tumorigenic M2-like to pro-inflammatory M1-like macrophages via activation of the ferroptosis pathway. APOC1 expression was found to be negatively correlated with programmed death-1 receptor (PD-1) and its ligand (PD-L1) in human HCC samples, suggesting new insights for anti-PD-1 immunotherapy in HCC.

An analysis of ten chronic HBV/HCV carriers with HCC has identified high inter-tumoral heterogeneity of macrophages, with some macrophage clusters being specifically associated with individual patients. Overall, eleven clusters were identified, of which most were enriched in the tumor, except for a macrophage receptor with collagenous structure (MARCO)+ cluster enriched in the non-tumor liver. Furthermore, by analyzing two additional HCC cohorts, a higher abundance of MMP9+ TAMs was found in tumors that were strongly associated with worse overall survival. The authors demonstrated the crucial role of PPARγ in the terminal differentiation of MMP9+ TAMs in HCC. Of the transcription factors analyzed, PPARγ was the most upregulated in this specific TAM subset. In addition, PPARγ affects HCC progression by controlling MMP9+ TAM differentiation, based on PPARγ inhibition assays in THP-1 cells. [104].

By integrating spatial transcriptomics with scRNA-seq and multiplexed immunofluorescence, researchers revealed a distinct spatial niche formed by SPP1+ macrophages and CAF at the tumor margin of anti-PD-1-treated HBV-HCC patients. Preclinical studies demonstrated that targeting this niche, either through SPP1 blockade or macrophages-specific Spp1 deletion, improved anti-PD-1 response in murine liver cancer models. These interventions reduced CAF accumulation while promoting intratumoral cytotoxic T cell infiltration [105].

A comprehensive analysis of the TME from ten HBV-associated HCC samples showing microvascular invasion (MVI) identified four distinct subsets among the macrophage population. The TREM2 macrophage subset, expressing apolipoprotein E and C1, exhibited features similar to lipid-associated macrophages (LAM) and was preferentially enriched in tumors with MVI. Metabolic pathway analysis demonstrated pronounced dysregulation in TREM2+ macrophages, suggesting their metabolic reprogramming may fuel tumor progression. Using CellChat to investigate putative cell–cell interactions, TREM2 macrophages were identified as key mediators of tumor progression via the midkine–nucleolin signaling axis. These findings indicate that malignant cells may actively recruit TREM2 macrophages into the TME, thereby promoting a more aggressive tumor phenotype [106].

Portal vein tumor thrombosis (PVTT) exacerbates the prognosis of HCC by promoting intrahepatic spread and contributing to portal hypertension. Recent CyTOF analysis demonstrated that macrophages and monocytes were the predominant immune cell populations within PVTT, present at higher proportions than in primary tumor tissue or peripheral blood. ScRNA-seq of samples from six patients with HBV-associated HCC revealed that TAM enriched in PVTT segregated into five distinct subpopulations, with the TAM-C5AR1 cluster emerging as the predominant subset. This population was marked by gene signatures associated with leukocyte chemotaxis and was implicated in PVTT pathogenesis through the creation of an immunosuppressive niche, correlating with poor clinical outcomes [107].

The two major types of primary liver cancer, HCC and iCCA, differ in morphology, metastatic capacity and characteristics of their immune microenvironment. A combined single-cell immune atlas of HCC and iCCA, constructed by integrating scRNA-seq analysis from different tissue types of HCC and iCCA, has made it possible to characterize the distinct immune microenvironments of the two tumors. Globally, higher fractions of myeloid cells were retrieved from samples of patients with HCC than from those with iCCA. Several myeloid subpopulations were identified, which were differentially distributed within the two cancers. Specifically, CD14+ monocytes were more frequently identified in iCCA than in HCC tumor samples. In contrast, higher fractions of EGR1+ macrophages and MARCO+ macrophages were observed in HCC tumors than in iCCA. In addition, the frequencies of these subpopulations in each individual patient were found to be different between the two types of tumors, indicating significant heterogeneity [108].

iCCA exhibits a characteristically dense desmoplastic microenvironment enriched with stromal, endothelial, and diverse immune cell populations. The immune landscape of iCCA remains poorly understood. ICCA is poorly infiltrated by immune cells and is commonly classified as an immunologically ‘cold’ tumor [109]. ScRNA-seq analysis of iCCA revealed heterogeneous TAM populations and identified a role for the S100A gene family. Macrophages segregated into four distinct clusters, with the predominant population exhibiting high expression of S100P and high levels of S100As genes (S100A4, S100A8, and S100A9) [110]. Notably, in colorectal cancer, S100A8/S100A9 upregulation in myeloid cells has been shown to drive their differentiation into myeloid-derived suppressor cells and M2-like macrophages [111].

In iCCA, M2-like macrophage infiltration was significantly increased in peritumoral compared with intratumoral areas and normal liver, as assessed by CD163 and CD206 expression [112]. The analysis of 6 spatial transcriptomic samples and 35 single-cell samples of iCCA revealed two distinct immune infiltration patterns, one of which was characterized by a predominance of CD68+MARCO+ macrophages and was associated with a poorer outcome. Spatial transcriptomics revealed the co-localization of MARCO+ TAMs with cathepsin E (CTSE+) tumor cells. High infiltration of both MARCO+ TAMs and CTSE+ tumor cells correlated with the poorest survival outcomes [113].

The liver flukes *Opisthorchis viverrini* and *Clonorchis sinensis* are well-established risk factors for CCA [114,115]. Etiology appears to shape the cellular composition of the TME in iCCA. Notably, comparisons between *C. sinensis*-associated and non-*C. sinensis* iCCA samples revealed distinct distribution patterns of immune cell subtypes between the two groups. In *C. sinensis* infection-related iCCA, CXCL10⁺ macrophages, MKI67⁺ macrophages, and SPP1-expressing macrophages were markedly enriched, creating a distinctive microenvironment specific to this etiology [116].

High-dimensional single-cell technologies were employed to characterize the T-cell and myeloid cell compartments in iCCA tissues, with comparative analyses of matched tumor-free peritumoral tissues and circulating immune cell populations. Tumor tissues exhibited a similar frequency of myeloid cells compared to adjacent peritumoral tissues. Within the myeloid compartment, two clusters of monocytes were identified: non-classical monocytes, expressing FCGR3A, CDKN1C, LILRA1, and LILRB2, and CD14^high^ classical monocytes, characterized by S100A8, VCAN, and CD36 expression. Additionally, three macrophage clusters were observed: the ID3^high^ macrophages expressing VSIG4 and resembling KC; MARCO^high^ macrophages expressing PLIN2, APOC1 and SPP1; and TREM2^high^ macrophages expressing APOC1 and C1QA/B/C. Interestingly, MARCO^high^ myeloid cells were increased in tumoral compared with peritumoral tissues [117].

Overall, these findings demonstrated that the TAM compartment in primary liver cancers is highly dynamic and remarkably heterogeneous, not only between patients but also among different malignant lesions. It is noteworthy that part of such heterogeneity reflects the ability of TAMs to acquire an entire spectrum of phenotypic, metabolic, and functional profiles in response to environmental stimuli.

## 5. Lipid Metabolism Reprogramming in TAM of Primary Liver Cancers

An increasing number of observations have demonstrated that macrophages display extraordinary plasticity in adapting to the local microenvironment. Similarly, TAMs in the TME reprogrammed their metabolic pathways in order to adapt to the special environment and to compete with other cells for nutrient scarcity. The extraordinary heterogeneity of TAM phenotypes and functions is also influenced by their own cellular metabolism. Generally, M1-like macrophages rely mainly on glycolytic metabolism, display impaired oxidative phosphorylation (OXPHOS), and synthesize FA from acetyl-CoA to obtain inflammatory mediators. Conversely, M2-like macrophages manifest increased FAO and OXPHOS, which has been associated with their ability to support tissue repair. Although consensus indicates a dichotomous model for M1- and M2-like activation, more data suggest that TAMs may be metabolically shaped by both glycolysis and OXPHOS in the TME [118,119,120].

Among the metabolic alterations frequently observed in TAMs, dysregulated lipid metabolism is a feature of TAMs that profoundly influences their function. The protumor phenotype exhibited by TAMs is driven by oxidation of arachidonic acid, which generates immunosuppressive metabolites like prostaglandin E2 and 15-hydroxyeicosatetraenoic acid, as well as by increased cholesterol efflux, mediated by the lipid transporter ABCA1 (ATP-binding cassette transporter A1) and signaling pathways such as IL-4/STAT6 and phosphatidylinositol 3-kinases [120].

FABPs are a family of lipid chaperones that reversibly bind saturated and unsaturated long-chain FAs, facilitating their transport to various cellular compartments and thereby modulating FA distribution and metabolism [121,122]. In patients with HCC, FABP proteins, including FABP1 and FABP5, have been identified as key regulators of TAM lipid metabolism, contributing to the acquisition of pro-tumorigenic characteristics. A scRNA-seq atlas reveals an FABP1-dependent immunosuppressive environment in HCC, with FABP1 found to be overexpressed in TAMs from stage III HCC tissues compared to stage II. FABP1 promotes an M2-like polarization state in TAMs through the PPARγ/CD36-mediated potentiation of FAO. In vitro experiments demonstrated that FABP1 inhibition drives macrophage repolarization from immunosuppressive M2-like to immunostimulatory M1-like phenotype, concurrently reducing PD-L1 expression and impairing HCC cell proliferation [123]. Proteomic profiling of monocytes from HCC tissues identified FABP5 as a key regulator of immune tolerance in HCC patients by reducing FAO in TAMs. This reduction leads to LD accumulation through suppression of the PPARα pathway and ultimately affects PD-L1 expression on Tregs. Furthermore, infiltration of FABP5⁺CD68⁺ cells has been linked to HCC progression in humans [124]. Emerging evidence reinforces the contribution of FABP5 to TAM metabolic and functional regulation. In their study, Yang X. et al. [125] utilized scRNA-seq data from HCC patients to establish elevated FABP5 expression in TAMs. Furthermore, they identified a distinct subset of FABP5⁺ lipid loaded macrophages in both human HCC specimens and experimental murine HCC models. Long-chain unsaturated FAs released by tumor cells were found to activate PPARγ via FABP5, conferring immunosuppressive properties on TAMs. FABP5⁺ macrophages upregulated lipid metabolic pathways while suppressing immune activation programs, reinforcing their immunosuppressive role in human HCC. Experimental studies revealed that FABP5⁺ lipid loaded macrophages impair T-cell and cytotoxic T lymphocyte activation, attenuating immunotherapy outcomes. Furthermore, HCC patients with high FABP5 expression had poorer overall survival rates [125]. The involvement of FABP5 in macrophage immune reprogramming was further validated using a choline-deficient high-fat diet mouse model that mimics obesity-associated HCC, with subsequent confirmation in human HCC tissue samples. FABP5 exhibits a dual role in HCC progression through cell type-specific mechanisms. Highly expressed in both cancer cells and anti-inflammatory macrophages [126], FABP5 inhibition promotes lipid peroxidation-induced ferroptosis in tumor cells while reprogramming TAMs toward a proinflammatory phenotype. In TAMs, FABP5 blockade upregulates CD80/CD86 expression, enhancing T cell proliferation and cytotoxicity.

Overall, these data show that in the interaction between HCC cancer cells and TAM, the expression of FABPs, particularly FABP5, and the exchange of lipid metabolites like long-chain unsaturated FAs, reprogram TAMs via the FABP/PPAR pathway. This process plays a key role in inhibiting antitumor T-cell immunity and promoting tumor immune escape. Along the same line, others have highlighted the role of tumor-derived long-chain FAs, carried by EVs, in regulating metastasis-associated macrophage (MAM) function in the liver. CD36 was upregulated in MAMs and it was responsible for the internalization of tumor-derived lipids that subsequently drove the metabolic and functional reprogramming of macrophages towards a M2-like phenotype [127].

A multi-omics analysis of the tumor tissue of 113 patients with non-viral HCC showed that HCC emerging in the setting of MASLD, which accounts for 23% of non-viral HCC cases, was characterized by an immune cell-enriched, exhausted TME characterized by M2 macrophage infiltration. In a lipidomics-based total FA profiling, it was found that palmitic acid levels were significantly higher in steatotic HCC samples than in non-steatotic HCC tissues. The authors showed that palmitic acid was responsible for the immunosuppressive phenotype of macrophages [128].

Another study linked the immunosuppressive phenotype of macrophages to their lipid content [129]. Mass spectrometry-based lipidomic analysis revealed the enrichment of several species of triglycerides and some species of diacylglycerols in monocytes treated with tumor-derived supernatant. At the molecular level, TNFα enabled monocytes/macrophages to internalize tumor-derived lipids, driving triglyceride synthesis and LD formation. The study revealed that LD-laden macrophages (LLMs) accumulate in HCC and play a key role in establishing an immunosuppressive TME. These findings provide preclinical evidence to support the development of therapies targeting LLMs in HCC patients.

Recent studies have identified transmembrane protein 147 (TMEM147) as a novel player in HCC tumorigenesis and macrophage metabolic reprogramming. In HCC, TMEM147 interacts with 7-dehydrocholesterol reductase, disrupting cholesterol homeostasis through the accumulation of downstream oxysterol 27-hydroxycholesterol (27HC). HCC cells secrete 27HC into the TME, where it directly modulates macrophage metabolism. This study reveals that cholesterol homeostasis disruption in HCC induces lipid accumulation in macrophages, triggering their metabolic reprogramming and driving M2-polarization of TAMs [16]. A separate investigation established a connection between cholesterol regulation and TAM functionality in HCC [130]. Researchers observed the upregulation of the cholesterol transporter ABCA1 in TAMs, which correlated with reduced intracellular cholesterol stores and elevated serum cholesterol levels. These findings further strengthen the rationale for developing LLM-targeted therapeutic strategies in HCC. Elegant in vitro experiments demonstrated that cholesterol efflux restricts macrophage differentiation and induces the generation of immunosuppressive TAMs. A further study showed that cholesterol metabolism shapes TAM metabolism and phenotype [131]. Direct cell–cell contact between the HCC cancer cell line and CD14+ monocytes in spheroid cultures increased the expression of CD206 and CD163 M2 markers. Monocyte transcriptome analysis showed an enrichment in cholesterol efflux and cholesterol metabolism pathways driven by the LIPA, LPL, APOE, LDLR, NR1H3, ABCA1, and CETP genes. The authors demonstrated that monocytes take up lipids and transfer them to lysosomes, where the lysosomal acid lipase (LAL) mediates lipid hydrolysis to yield free FAs and free cholesterol. The inhibition of LAL affected the phenotype of monocytes, reducing M2 markers and CD36 expression, resulting in lipid accumulation.

Hepatic stellate cells (HSCs), comprising approximately 10% of the liver’s resident cell population, occupy a strategic position in the subendothelial space of Disse. Situated between hepatocytes and sinusoidal endothelial cells, these specialized cells play pivotal roles in liver pathology, particularly in cirrhosis and HCC development. Quiescent HSCs contain numerous specialized LDs that serve as the primary hepatic reservoir for retinoids (vitamin A and its metabolites). LDs additionally store diverse lipid species, including triglycerides, cholesterol esters, free FAs, and phospholipids [132]. HSCs undergo profound phenotypic transformation upon liver injury or during in vitro culture, transitioning from quiescent vitamin A-storing cells to activated myofibroblasts. This activation process involves the loss of characteristic LDs and the acquisition of a proliferative, contractile phenotype. Activated HSCs gain enhanced capacity for ECM production while developing pro-inflammatory and chemotactic properties [133]. Activated hepatic stellate cells (aHSCs) modulate immune responses by secreting cytokines, chemokines, and retinoids. Notably, a recent study has shown that a distinct population of CX3CR1+Ly6C+ TAM migrates to and interacts with aHSCs in the peritumoral region. In this niche, retinoids upregulate arginase-1 expression in macrophages, leading to arginine depletion and subsequent impairment of CD8+ T cell function—thereby promoting HCC progression [134].

Collectively, these studies have established that a range of tumor-derived lipids and distinct lipid transporters regulates macrophage lipid metabolism, ultimately driving the acquisition of an immunosuppressive phenotype.

In contrast, few studies on CCA have explored the impact of lipids on macrophage metabolism and function. In iCCA patients, overall survival and response to immunotherapy were significantly linked to *C. sinensis* infection. FA biosynthesis and the expression of FASN were notably enriched in *C. sinensis*-related iCCA. The observed metabolic reprogramming in tumor cells correlated with increased TAM infiltration and suppressed T-cell activity, collectively establishing an immunosuppressive niche that promoted tumor progression. Spatial transcriptomic profiling additionally demonstrated closer physical interactions between malignant cells and TAMs in *C. sinensis*-related iCCA compared to non-*C. sinensis*-related iCCA [116].

Another study revealed by immunohistochemical analysis that 5-lipoxygenase (ALOX5), an enzyme involved in the synthesis of lipid mediators, was significantly upregulated in iCCA tissues. ALOX5 expression in iCCA cells influenced the infiltration of M2 macrophages into the TME. Molecular analysis identified LTB4 as a key driver of iCCA progression through its ability to recruit M2 macrophages [135]. The ALOX5 metabolite achieves this by activating BLT1/BLT2 receptor-dependent PI3K signaling [135].

In our previous work, we demonstrated that EVs isolated from the supernatants of primary iCCA cells displayed an altered sphingolipid profile, which influences monocyte function by promoting the secretion of pro-inflammatory cytokines, a process mediated by ceramide [51]. Subsequently, we investigated the influence of tumor cells on the lipid content of the TME by co-culturing primary iCCA cells with the THP-1 monocyte cell line. Notably, THP-1 cells exhibited increased lipid accumulation, higher expression of the CD36 scavenger receptor, and elevated M2 markers (CD163 and CD11b). THP-1 co-cultured with primary iCCA cells determined a lower activation and proliferation of T cells, suggesting that lipids harness the phenotype of THP1 cells towards immunosuppression [10].

## 6. Conclusions

**Figure 2:**
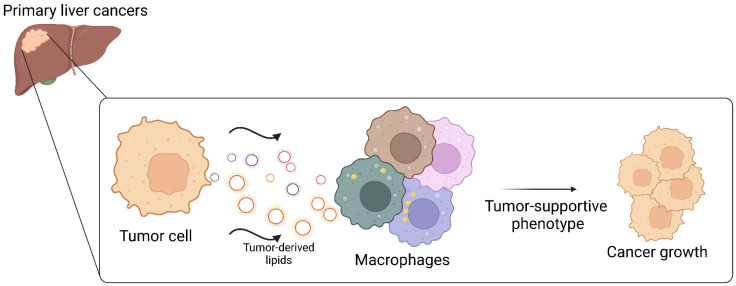
The lipid content and lipid metabolism of tumor cells are profoundly altered in primary liver cancers, perturbing the tumor microenvironment. Recent advances in single-cell and spatial technologies have revealed that macrophages within the tumor microenvironment of primary liver cancers constitute a remarkably heterogeneous population, characterized by diverse phenotypes, multifaceted functions, and a high degree of plasticity. Macrophages are influenced by the exchange of lipids derived from tumor cells, which ultimately promote immunosuppression and tumor progression. This presentation was created by BioRender (version 201).

Thanks to recent advances in single-cell and spatial techniques, various studies have demonstrated that in the TME of primary liver cancers, TAMs represent an extremely heterogeneous population with diverse phenotypes, functions, and high plasticity (Figure 2). It has been established that the lipid content and lipid metabolism of tumor cells undergo profound alterations, leading to a significant transformation of the TME. As a result, TAMs are modulated by the exchange of metabolites and lipids derived from tumor cells, which ultimately drive immunosuppression and facilitate tumor progression. Such altered pathways offer valuable insights to identify druggable targets, providing opportunities to test novel therapeutic interventions.
